# The Sugar Ration

**Published:** 1917-03-31

**Authors:** 


### FOOD IN WARTIME.*

### The Sugar Ration.

In days when sugar was cheap and plentiful a pound was the ordinary allowance per head per week, and this was considered about sufficient for beverages alone, in addition to what might be used in cooking. Accordingly, when the edict went forth that only ¾ lb. could be spared a head for all purposes, cooking and drinking together, a good many housekeepers denounced the ration as insufficient. However, it must be made to cover all needs.

Sugar is one of those articles which are used collectively, and it will prove most convenient to reckon out the whole bulk to which the numbers resident in an institution are entitled, and practise each week to make this amount last out. Patients and staff are much on the same footing in this particular, and low diets, do not differ greatly from full diets. For all must drink, and whether it be tea, or coffee, or cocoa, or lemonade, or barley water, sugar is a common ingredient.

Taking the case of the patients first, it will be found, we believe, that only a very small proportion are in the habit of drinking tea or coffee unsweetened. To alter their habits at a time when they have so much to endure in other ways would be a very inadvisable proceeding, and should not be attempted. They get, as a rule, tea or some such beverage twice a day, and about a pint, or two cups, each time. The tea, whether made in bulk or in teapots, will require 1 oz. of sugar to a quart, or a flat teaspoonful to a half-pint cup. It is clear, therefore, that each patient drinks up about 7 oz. of sugar a week. This leaves 5 oz. more out of his ration to be taken in the form of pudding. Half an ounce of sugar (two flat teaspoonfuls) is quite sufficient to sweeten the ordinary portion of pudding, and thus the patient is left with 1½ oz. over when the week is done; not too much, perhaps, to allow for heavy-handedness on the part of those responsible for weighing out the quantities. It is quite possible that the quantities we have estimated may not produce the syrupy flavour in the tea approved by some connoisseurs. But the point now is to live within the Government ration, not to gratify in every particular palates spoiled by over-cheap supplies. There are many degrees of sweetness, and everyone must be content with what he can get. The proportion of a full ounce to a quart yields a very fair result.

The sugar ration of the staff is affected by the fact that a very large section of nurses take no sugar in tea, and many who have always hitherto been accustomed to it have taught themselves to do without it since it became so scarce. The existence of the "sugar hog," however, is a nuisance which cannot be ignored. There will always be people in whom deprivation of any sort awakens selfishness and greed, and it would seem we may be returning to the days of our great-grandmothers when sugar was a coveted delicacy to be locked in the centre compartment of the tea-caddy. As sweet things of any description have now become more rare, it might perhaps be a just plan to issue an extra jam ration to such as do not take sugar in their tea, and if this should have the effect of increasing the number of abstainers, all the better. Undoubtedly the sugar ration goes farther and has more effect when introduced into articles of diet than when used to sweeten a beverage. It is ample to provide puddings daily, with cakes occasionally, and may easily be made to stretch to a supper dish if the above precautions in regard to beverages are observed.

In one particular the ration may run short. Fruit of all kinds, when cooked, requires a great deal of sweetening, not only in the cooking, but also on the table. The use of a small quantity of carbonate of soda in cooking is advocated to neutralise the acid. If this expedient be not approved, it will be found that a little milk poured on the fruit instead of cream softens the flavour. Without some such aids the rhubarb season may prove inopportune, and the gooseberry will be less welcome than usual. We would suggest that the weekly allowance for the household be totalled up once a week, and the surplus used to sweeten the very necessary fruit tarts and puddings. If a little wholesome parsimony be exercised over the beverages, there should be enough sugar over to meet these calls.

The important matter is to take the staff into confidence. Let all help to solve the problem. Let everyone know each week how the average consumption of sugar for the household stands, so that, if necessary, increased economy may be enforced. The lazy attitude adopted by some institution housekeepers who base their obedience to national demands merely on hopeful estimates, and are content with using "as little as possible" instead of working close to the ration, is entirely indefensible.

* Previous articles appeared on February 24, March 10, and March 24.

